# The role of dynein mediated transport in the clearance of misfolded proteins responsible for motoneuron diseases

**DOI:** 10.1186/2193-1801-4-S1-L24

**Published:** 2015-06-12

**Authors:** Riccardo Cristofani, Elisa Giorgetti, Valeria Crippa, Alessandro Boncoraglio, Marco Meroni, Mariarita Galbiati, Paola Rusmini, Elio Messi, Angelo Poletti

**Affiliations:** Università degli Studi di Milano, Italy

**Keywords:** MNDs, PQC, dynein

Spinal and bulbar muscular atrophy (SBMA) and amyotrophic lateral sclerosis (ALS) are motoneuron diseases. A mutation in the androgen receptor (ARpolyQ) gene is responsible for SBMA. Mutations in the SOD1, in the TDP-43, in the FUS-TLS or in the C9ORF72 genes are responsible for familiar form of ALS. The mutated coded proteins misfold and aggregates. Efficient protein quality control (PQC) is required for the maintenance of physiological and soluble protein pool in affected motoneuron. The balance between autophagy and ubiquitin-proteasome system (UPS) prevents protein aggregation and increases degradation of SBMA and ALS misfolded proteins. Dynein binds the co-chaperone BAG3 and transports the mutant proteins at microtubule organization center where misfolded proteins interact, aggregate and can be degraded by autophagy. However, here misfolded proteins may blocks autophagy flux. In NSC34 cells, dynein is sequestered into ARpolyQ aggregates suggesting the role of dynein into aggregates formation process. Unexpectedly, the silencing of dynein heavy chain resulted in a drastic reduction of ARpolyQ retained in filter retardation assay (FRA). Moreover, dynein silencing drastically altered autophagic markers localization (LC3 and p62) by immunofluorescence. Notably, treatment with a dynein inhibitor (EHNA) drastically reduced the retention of ARpolyQ, mutSOD1 and mutTDP43 aggregates in FRA, even when autophagy was inhibit with 3-MA. Conversely UPS blockage with MG132 counteracted the reduction induced by altered dynein transports. RTq-PCR on NSC34 cells treated with EHNA showed an increased BAG1:BAG3 ratio that can targeting the misfolded proteins to UPS. Moreover, in NSC34 cells, EHNA increased the degradation of proteasome reporter GFPu, while BAG1 overexpression reduced the level of aggregates retained in FTA. These data suggest that, when autophagy is overload, by misfolded proteins, dynein inhibition restores the physiological and soluble protein pool via UPS.

